# Changes in Gonadal Sex Differentiation, Digestive Enzymes, and Growth-Related Hormone Contents in the Larval and Juvenile Black Scraper, *Thamnaconus modestus*

**DOI:** 10.3390/biology14101385

**Published:** 2025-10-10

**Authors:** Wengang Xu, Yan Liu, Jiulong Wang, Pei Yang, Yanqing Wu, Liming Liu

**Affiliations:** 1 School of Ocean, Yantai University, Yantai 264005, China; liuyansd@ytu.edu.cn (Y.L.); wangjiulong@ytu.edu.cn (J.W.); peiyang1909@163.com (P.Y.); 2 Yantai Engineering Research Center of Deep-Sea Aquaculture of Economic Fish, Yantai 264005, China; 3 Shandong Engineering Research Center of Healthy Land-Sea Relay Farming of Economic Fish, Yantai 264005, China; 4 East China Sea Fisheries Research Institute, Chinese Academy of Fishery Sciences, Shanghai 200090, China; wuyanqing0961@163.com

**Keywords:** *Thamnaconus modestus*, sexual differentiation, estradiol, testosterone, digestive enzymes, growth-related hormones

## Abstract

To understand the changes in gonadal sex differentiation, digestive enzyme activity, and growth-related hormone levels in the larval and juvenile black scraper, *Thamnaconus modestus*, continuous sampling was conducted from 0 to 91 days post-hatching (dph). 17β-estradiol (E2) and testosterone (T) levels, six digestive enzymes, as well as T3, T4, GH, and IGF-I were detected. The results showed that oogonia or spermatogonia was observed at 60 dph. During the sex differentiation to female or male, both E2 and T levels significantly increased (*p* < 0.05), suggesting that E2 and T may induce the sex differentiation to female or male in *T. modestus*, respectively. The amylase activity from 0 to 35 dph showed a slow upward trend, which may be due to the transition from endogenous to exogenous nutrition at this time. From 12 to 25 dph, alkaline protease activity significantly decreased (*p* < 0.05), while acid protease levels significantly increased (*p* < 0.05), suggesting that as organs in the digestive system continue to develop, acid protease plays an important role. T3 and T4 could already be detected at 0 dph, and the T4 content was always much higher than T3 throughout the stages, indicating that T4 may play more important roles than T3. Additionally, the changes in IGF-I and GH content followed a trend of an initial increase, a subsequent decrease, and then an increase, ultimately showing an overall upward trend. These results indicate that T4, IGF-I, and GH play crucial roles in growth and development in the juvenile fish. In conclusion, the results of this study provide useful information for growth, artificial reproduction, and sex regulation in *T. modestus*.

## Simple Summary

Sex hormones, digestive enzymes, and growth-related hormones play important roles in the early phases of marine fish including sexual differentiation, growth, and development. This study aimed to understand these changes in the larval and juvenile black scraper, *Thamnaconus modestus*. 17β-estradiol (E2) and testosterone (T) contents were detected, as well as the activities of digestive enzymes and the levels of Triiodothyronine (T3), thyroxine (T4), growth hormone (GH), and insulin-like growth factor-I (IGF-I). The results suggest that the gonads had differentiated into ovaries or testes at 60 dph in *T. modestus*, perhaps due to increased estrogen and androgen levels, respectively. The activity of six digestive enzymes could be detected in newly hatched larvae, suggesting that the larvae already have the ability to digest food. Furthermore, T3 and T4 could be detected as early as at 0 dph, and the T4 content was always much higher than that of T3 in each stage, indicating that T4 may play more important roles than T3. The levels of IGF-I and GH are positively correlated with development in fish, suggesting that IGF-I and GH jointly promote fish growth. This study may be beneficial to promote the artificial reproduction and healthy growth in *T. modestus*.

## 1. Introduction

Gonadal sex differentiation is an important stage in the early growth and development process of fish. Research indicates that the gonads of fish are primarily formed through the division, migration, and differentiation of somatic cells derived from the body cavity membrane and primordial germ cells (PGCs) [1,2]. After PGCs migrate to the reproductive ridge via the mesoderm or blood circulation, the reproductive ridge continues to develop and extend forward into the primordial gonads [3,4]. Furthermore, research on various fish species has shown that sex steroid hormones are important inducers of gonadal differentiation, such as endogenous estradiol (17β-estradiol, E2) and testosterone (T), which can effectively induce undifferentiated gonads to differentiate into ovaries or testes [5], as reported in the red spotted grouper *Epinephelus akaara* [6] and tilapia *Oreochromis niloticus* [7].

Furthermore, in the early stages, the digestive systems of most marine fish are in an undeveloped state, and protein digestion is limited to trypsin, with the main digestion method being endocytosis. As development progresses, acid-secreting cells in gastric glands begin to adopt secretion functions, and gastric protease becomes the main digestive enzyme for proteins [8]. The appearance of alpha amylase, produced by zymogen granules secreted by pancreatic acinar cells, is considered a marker of pancreatic cell differentiation and maturation in juvenile fish [9]. In rapidly developing marine fish, the amylase activity exchange pattern usually increases rapidly before initial feeding, followed by continuous fluctuations [10]. Lipase is a water-soluble enzyme that catalyzes the hydrolysis of ester bonds in lipids, playing a crucial role in the digestion, transportation, and cleavage of lipids such as triglycerides, fats, and oils. Acid and alkaline phosphatases are widely present in various animal bodies, catalyzing the transfer and metabolism of phosphate groups under acidic and alkaline conditions, respectively [11].

The hormones such as triiodothyronine (T3) and thyroxine (T4) secreted by the endocrine system also play important roles in the early growth, development, survival, and metamorphosis of juvenile fish [12,13,14]. In addition, thyroid hormone can enhance the adaptability of fry to different environmental changes, induce synchronous growth, and improve digestive function [15]. Growth hormone (GH) promotes growth through both direct and indirect mechanisms. Direct action refers to GH directly acting on GH receptors on target cells, stimulating cell differentiation through GH-related signaling pathways. Indirect effects refer to the promotion of growth by inducing the production of insulin-like growth factor-I (IGF-I) [16].

The black scraper *Thamnaconus modestus*, commonly known as the skinny wolf, skin-flaying fish, and pufferfish in China, belongs to the order Tetraodontiformes, family Monacanthidae, and genus Thamnaconus. It is a warm-water and near-bottom fish species [17]. From the 1960s to the 1980s, *T. modestus* was a major marine fish economically in China, with annual catches second only to the hairtail, *Trichiurus lepturus*. Furthermore, the annual catch of *T. modestus* was 2,300,000 tons in the 1980s in Korea [18]. Since the 1990s, due to increased market demand, overfishing, and the lack of successful artificial breeding technology, its resource population has declined sharply [19]. In 2024, the wild catch of *T. modestus* was only 119,172 tons [20]. Therefore, it has been listed as an internationally endangered species [21]. In order to maintain market demand and the ecological balance of natural resources, there is an urgent need for large-scale artificial breeding. However, there are currently few research reports on gonadal differentiation and changes in endocrine hormone levels after hatching, which limits the development of artificial breeding, seed hatching, and aquaculture technology.

Research has shown that the weak activity of digestive enzymes during the early development stage is a key factor in the high mortality rate of fish fry [22]. Moreover, during the early development process of fish, as their digestive system continues to improve, there are significant differences in digestive enzyme activity at different stages, which also have an important impact on the growth and survival rates of fish fry [23,24]. Fish hormones, especially thyroid and growth hormones, also play important regulatory roles in the early stages of fish life history [25,26]. However, their roles and function after hatching have remained unclear in *T. modestus*. Furthermore, few studies have focused on the regulatory mechanism of sexual differentiation in these stages. These unknown factors limit the growth, development, and reproduction during the early life stage in *T. modestus*. Therefore, this study aimed to investigate the changes in gonadal differentiation mechanism, digestive enzymes, and growth-related hormone content in the larval and juvenile *T. modestus*. The results in this study may provide useful information for artificial reproduction and healthy growth in this species.

## 2. Materials and Methods

### 2.1. Experimental Materials

The *T. modestus* eggs used in the experiment were obtained from Yantai Pusheng Aquatic Products Co., Ltd. (Yantai, China) through the natural mating of male and female parent fish. On 10 April 2023, the hatched fry were placed in a 20 m^3^ cement pool for open-water aquaculture, with a pH of 7.6–8.1, salinity of 28–34, aeration for 24 h, and the dissolved oxygen maintained above 5 mg/L. During the experiment, the water temperature changed between 19.5 and 22.0 °C. S-type rotifers were fed at 3–7 days post-hatching (dph), L-type rotifers at 8–20 dph, and brine worms at 18–35 dph. The domestication of compound feed began at 30 dph, and it was gradually fed in the later stages.

After hatching, samples were taken every 5 days with 30 individuals each time. Firstly, the fish were anesthetized with MS-222 (Hangzhou Animal Medicine Factory, Hangzhou, China), at the dosages prescribed for various teleosts [27] and a concentration of 70 mg/L. Then the body weight (BW), total length (TL), and body length (BL) were measured (Table 1) and gonads were sampled. Due to the small size of the gonads in newly hatched fish, 15 specimens were fixed as a group in Bouin’s solution in samples less than 2 cm in TL. After 24 h, they were transferred to 70% ethanol for histological analysis. The remaining 15 fish with the entire bodies were stored together in a −80 °C freezer for the determination of sex hormone levels, as they were too small for blood to be obtained. In samples larger than 2 cm in TL, the blood was obtained by capillary and stored in a −80 °C freezer, and the torsos were fixed in Bouin’s solution for histological analysis.

Samples were also taken at 0, 12, 25, 35, 50, 57, 64, 79, and 91 dph to detect the digestive enzymes and growth-related hormone levels. Before sampling, the sample was placed in seawater without bait organisms and feeding was stopped for 4 h. We collected a total of 2/3 of the volume of a 1.5 mL centrifuge tube at 0 and 12 dph. The collection volume for samples at 25 and 35 dph was about 2/3 of the volume of 5 mL centrifuge tubes, with a total of 3 centrifuge tubes collected each time. Fish fry after 35 dph should be sampled separately, with 9 individuals each time, and stored in a −80 °C freezer.

### 2.2. Experimental Methods

#### 2.2.1. Observation of Gonadal Histology

The samples stored in Bouin’s solution were subjected to gradient alcohol dehydration, paraffin embedding, sectioning, and staining with hematoxylin and eosin (Supplementary Material).

#### 2.2.2. Detection of Sex Hormone Content

The levels of E2 and T were detected using an enzyme-linked immunosorbent assay as described by our previous study [28] (Supplementary Material). Each sample was measured three times repeatedly.

#### 2.2.3. Detection of Digestive Enzyme Activity and Hormone Content

The whole fry at 0–25 dph and the abdomen at 30 and 35 dph were used for homogenization. A certain proportion of physiological saline was added. The samples were homogenized under ice–water bath conditions, and then placed in a freeze centrifuge. After centrifugation at 4 °C and 2500 r/min for 10 min, the supernatant was taken to detect the activity of various digestive enzymes (Supplementary Material). Each sample was measured three times repeatedly.

All experimental procedures involving animals were conducted in compliance with the Animal Care and Use Committee of Yantai University, China (Permit Number 20230605).

### 2.3. Data Analysis

The three replicates were used in each analysis. SPSS v26.0 software (IBM, Armonk, NY, USA) was used to compare and analyze the experimental data, and all data are expressed as means ± standard deviation (SD). The Kolmogorov–Smirnov method was used for normal distribution detection, and one-way ANOVA (analysis of variance) and Tukey’s HSD (honestly significant difference) were used for comparative analysis of differences. Bonferroni correction was used for multiple comparisons. *p* < 0.05 was considered significant.

## 3. Results

### 3.1. Sex Differentiation of Female and Male Juvenile Fish

**Figure 1:**
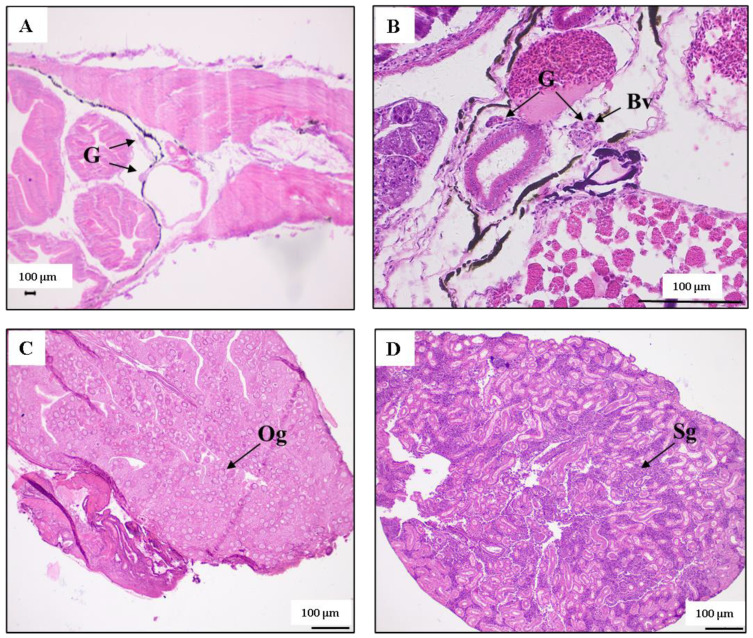
Histological observations of sex differentiation in the female and male *Thamnaconus modestus*. (**A**) 25 days post-hatching (dph); (**B**) 35 dph; (**C**) ovary at 60 dph; (**D**) testis at 60 dph. G, gonad; Bv, blood vessel; Og, oogonia; Sg, spermatogonia.

At 25 dph, undifferentiated gonads were observed, appearing in pairs near the intestine and connected to the mesentery (Figure 1A). At 35 dph, undifferentiated gonadal and blood vessels were observed (Figure 1B). At 60 dph, oogonia (Figure 1C) or spermatogonia (Figure 1D) was observed.

### 3.2. Changes in E2 and T Contents Before and After Sex Differentiation

**Figure 2:**
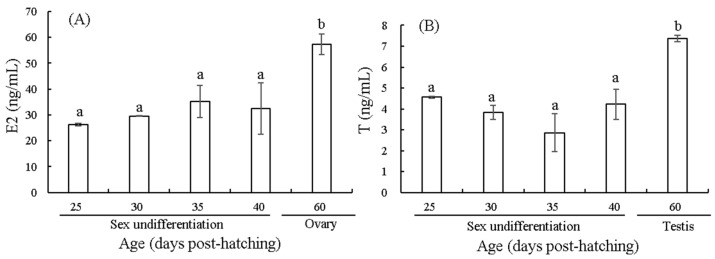
Changes in contents of E2 (**A**) and T (**B**) around sex differentiation in *T. modestus*. The 15 individuals were detected in each age. The data is shown as means ± SD. The different lowercase letters indicate significant differences (*p* < 0.05), while labels with the same or no letters indicate no significant differences (*p* > 0.05).

The E2 levels show an upward trend (Figure 2A). At 25 dph, the level is low; it shows an upward trend at 35 and 40 dph; and it significantly increased at 60 dph (*p* < 0.05). The T levels show a trend of first decreasing and then increasing (Figure 2B). At 25, 35, and 40 dph, the T levels decreased and then increased. At 60 dph, it significantly increased (*p* < 0.05).

### 3.3. Changes in Digestive Enzyme Activity of T. modestus

**Figure 3:**
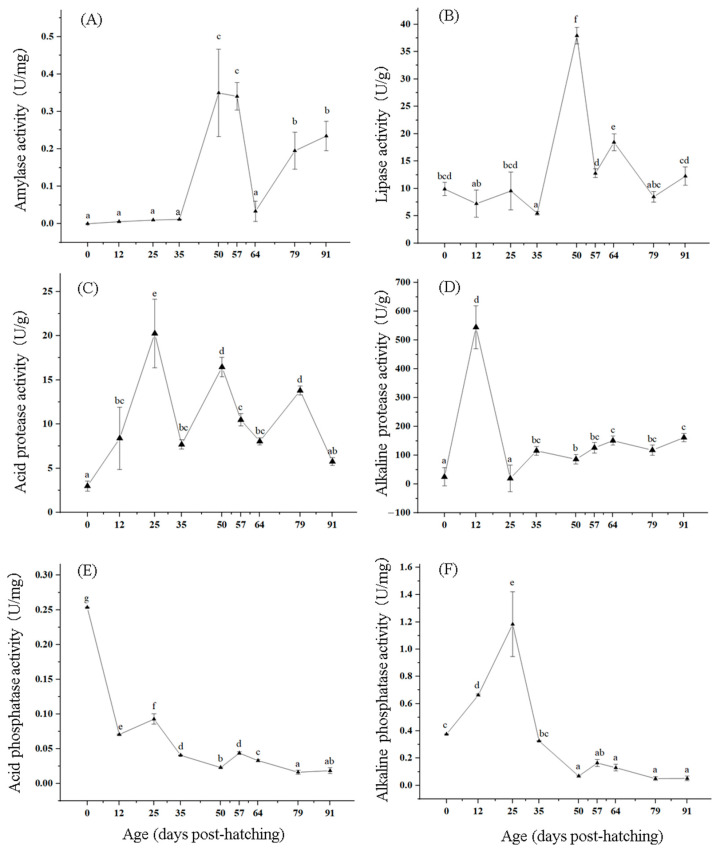
Changes in activities of amylase (**A**), lipase (**B**), acid protease (**C**), alkaline protease (**D**), acid phosphatase (**E**), and alkaline phosphatase (**F**) in *T. modestus* from 0 to 91 dph. The nine individuals were detected in each age. The data is shown as means ± SD. The different lowercase letters in the same line indicate significant differences (*p* < 0.05), while labels with the same or no letters indicate no significant differences (*p* > 0.05).

The amylase activity was the lowest at 0 dph, and then increased, reaching peak at 50 and 57 dph, respectively, which were significantly higher than at other ages (*p* < 0.05). Subsequently, it gradually decreased and reached a trough at 64 dph (Figure 3A).

The lipase activity was 9.91 ± 1.20 U/g at 0 dph, significantly decreased to the lowest point at 35 dph (*p* < 0.05), and significantly increased at 50 dph (*p* < 0.05), reaching a peak with 37.93 ± 1.49 U/g (Figure 3B).

The activity of acidic protease was the lowest at 0 dph, only 2.99 ± 0.58 U/g, and then increased. Peaks appeared at 25, 50, and 79 dph, all significantly higher than at other ages (*p* < 0.05). At 35, 64, and 91 dph, troughs appeared (Figure 3C).

The alkaline protease activity was 25.14 ± 3.15 U/g at 0 dph. Subsequently, it reached a peak at 12 dph, significantly higher than at other ages (*p* < 0.05). Subsequently, it significantly decreased and reached its lowest point at 25 dph (Figure 3D).

The activity of acid phosphatase was highest at 0 dph with 0.25 ± 0.01 U/mg, significantly higher than at other ages (*p* < 0.05). At 12 dph, it significantly decreased (*p* < 0.05), and then significantly increased (*p* < 0.05) at 25 dph. Subsequently, it decreased and reached a minimum of 0.0164 ± 0.0030 U/mg at 79 dph (Figure 3E).

The activity of alkaline phosphatase was relatively low at 0 dph, with 0.377 ± 0.002 U/mg, and significantly increased at 25 dph (*p* < 0.05), reaching a peak, followed by a significant decrease (*p* < 0.05), and it reached a trough at 50 dph. At 57 dph, it slowly increased and then decreased, reaching a minimum at 79 dph (Figure 3F).

### 3.4. Changes in Growth-Related Hormone Levels

**Figure 4:**
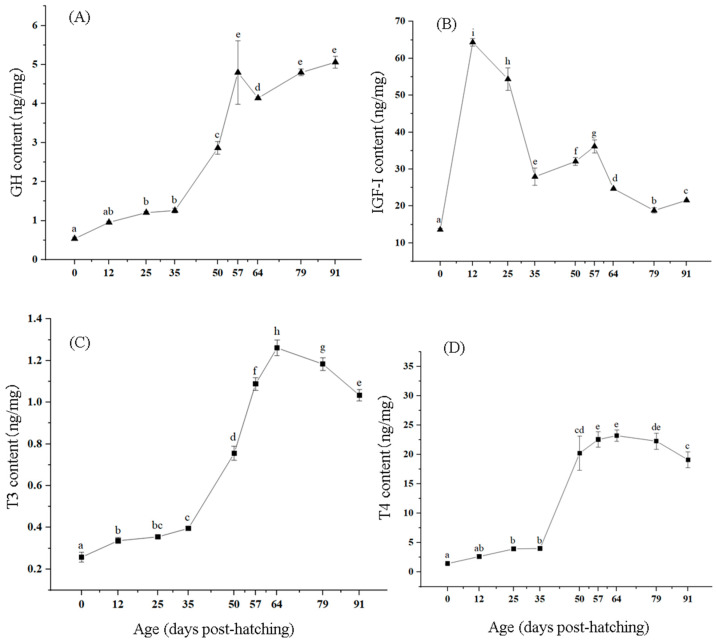
Changes in contents of GH (**A**), IGF-I (**B**), T3 (**C**), and T4 (**D**) in *T. modestus* from 0 to 91 dph. The nine individuals were detected in each age. The data is shown as means ± SD. The different lowercase letters in the same line indicate significant differences (*p* < 0.05), while labels with the same or no letters indicate no significant differences (*p* > 0.05).

GH was detected throughout the process, with a change in content that first increased, then decreased, and then increased again. At 0 dph, the GH content was the lowest, and then it rapidly increased. At 57 dph, the GH content reached a peak, significantly higher than at other ages except for 50 dph (*p* < 0.05). Afterwards, the GH content gradually decreased, and then it gradually increased until 91 dph (Figure 4A).

At 0 dph, the IGF-I content was the lowest, and then it rapidly increased, reaching a peak at 12 dph, significantly higher than at other ages except 50 dph (*p* < 0.05). Afterwards, the IGF-I contents gradually decreased, and it reached a trough at 35 dph. Subsequently, the content slowly increased at 57 dph, and then slowly decreased (Figure 4B).

Throughout the stages, both T3 and T4 were detected, and their trends were similar, showing an initial increase followed by a decrease, with T4 consistently higher than T3. At 0 dph, the minimum T3 content was detected, followed by a slow increase. It was not significant from 12 to 35 dph, but significantly increased from 35 to 50 dph (*p* < 0.05). T3 showed a peak at 64 dph, followed by a slow decrease until 91 dph (Figure 4C).

At 0 dph, the T4 content was the lowest. Subsequently, it gradually increased and reached the maximum at 64 dph, which was significantly higher than that at 0–50 dph (*p* < 0.05). Subsequently, it slowly decreased until 91 dph (Figure 4D).

## 4. Discussion

### 4.1. The Size and Timing of Sex Differentiation in T. modestus

Research has shown that, in bony fish such as the barfin flounder *Verasper moseri* [29] and Olive flounder *Paralichthys olivaceus* [30], the ovarian cavities have already formed at a total length of 35 mm and 15–30 mm, respectively, indicating that their gonads have differentiated into females. In addition, at the completion of sexual differentiation, the red spotted grouper *E. akaara* has a body length of 88.67 ± 2.18 mm and a body weight of 11.61 ± 0.59 mg, while the blacktip grouper *E. fasciatus* has a body length of 75.56 ± 1.95 mm and a body weight of 9.46 ± 0.44 mg [31]. The body length of the longtooth grouper, *E. bruneus*, is 77.9 ± 11.6 mm, and the body weight is around 9 mg [32]. In this study, *T. modestus* completed gonadal differentiation with a body length of 62.19 ± 0.05 mm and a body weight of 2460.56 ± 109.89 mg. This body length was longer than that of flounder but shorter than the grouper. In addition, in tiger puffer *Takifugu rubripes*, when gonadal differentiation is complete, the body length is approximately 33.62 mm [33]. The length of Yellowbelly pufferfish *T. flavidus* is 71.59–80.54 mm [34]. In this study, the body length of *T. modestus* during gonadal differentiation was between that of the *T. rubripes* and *T. flavidus*, indicating that, even though they belong to the same order of pufferfish, there are differences in body length during sexual differentiation.

In addition, studies have shown that the sexual differentiation of the red spotted grouper and blacktip grouper appeared at 80 dph and that the ovarian stage occurs at 120 dph [31]. In the malabar grouper *E. malabaricus*, the differentiation stage is at 74 dph and the ovarian stage is at 144 dph [35]. In this study, the gonads of *T. modestus* were undifferentiated at 35 dph, but gonadal differentiation was completed at 60 dph. It is speculated that the age of sexual differentiation into ovaries or testes in *T. modestus* is between 35 and 60 dph, which is shorter than that of the grouper, indicating that the age of sexual differentiation varies among different fish species. However, among fish of the same species, such as *T. rubripes*, the completion time for the ovaries and testes is at 47 and 61 dph, respectively [33]. In this study, the completion time for gonadal differentiation in *T. modestus* was close to or slightly later than that in previous research results. Furthermore, it was reported that water temperature plays an important role in the process of sex differentiation in the goldfish *Carassius auratus* [36] and European sea bass *Dicentrarchus labrax* [37]. Further studies should focus on the effects of water temperature on sex differentiation in *T. modestus*.

### 4.2. Effects of E2 and T on Sex Differentiation

Endogenous estrogen is critical in ovarian differentiation and maintenance [38]. In this study, the E2 content in female *T. modestus* was relatively low before differentiation. After differentiation, the E2 content significantly increased, indicating that E2 may be involved in inducing sex differentiation in the female fish. During the early stage of ovarian differentiation in *E. akaara*, estrogen plays an important role and may be related to the initiation of ovarian differentiation [11]. Estrogen plays a decisive role in the sex determination process in the Nile tilapia *O. niloticus* [39]. In the Eurasian perch *Perca fluviatilis*, the E2 content is low before gonadal differentiation, peaks in the late differentiation stage, and then shows a decreasing trend [40]. The role of E2 in the female differentiation process in *T. modestus* in this study was consistent with the above results. In this study, the T content of male *T. modestus* was relatively low before differentiation. After differentiation, the T content significantly increased, indicating that T may be involved in inducing male fish sex differentiation. A similar result was observed in *O. niloticus*, suggesting that androgens play an important role in maintaining the testis, particularly in the proliferation and differentiation of spermatogonia [41]. Therefore, this study suggested that E2 and T may, respectively, induce the gonadal differentiation in female and male *T. modestus*, and further studies should focus on the regulatory mechanism of two hormones in this species.

### 4.3. Effects of Digestive Enzymes on Growth and Development

During the early stages of fish development, as digestive organs form and improve, various digestive enzymes in their endocrine system undergo certain changes in different stages of growth [23,42]. In addition, changes in individual growth and digestive organ development [43], changes in feed types [44], and changes in enzyme expression caused by larval metabolism can all lead to changes in digestive enzyme activity [45]. The two key periods for quantitative changes in digestive enzyme activity are the period of transition from endogenous nutrition to exogenous nutrition in newly hatched larvae and the period of transition from larvae to juveniles [46]. In this study, it was found that the activity of acid protease, alkaline protease, acid phosphatase, alkaline phosphatase, amylase, and lipase could be detected in newly hatched larvae of *T. modestus*, suggesting that the larvae already have the ability to digest food when transitioning from endogenous to exogenous nutrition.

In this study, the amylase activity showed a slow upward trend from 0 to 35 dph, which may be due to the transition from endogenous to exogenous nutrition at this time. A similar result was reported in the leopard grouper *Mycteroperca rosacea* [47] and walleye pollock *Theragara chalcograma* [48], indicating that some marine fish already have the ability to digest carbohydrates in their early developmental stages. Cousin et al. [46] reported that the ingestion of exogenous nutrients such as rotifers and nauplius larvae may increase amylase activity. The amylase content significantly increased between 35 and 50 dph, possibly because the feed mainly consisted of nauplius and compound feed with a higher starch content. This is consistent with the view of Cahu et al. [9] that the starch content in artificial compound feed may also promote an increase in amylase activity in fish. However, the amylase activity significantly decreased from 50 to 64 dph, possibly due to a decrease in sugar demand as the fish fry grew and developed, leading to a decrease in amylase activity.

In this study, the lipase activity could be detected in various stages of *T. modestus*, and was also detected in the early development stages in the *T. chalcogramma* [48], common dentex *Dentex dentex* [49], and Mayan cichlid *Cichlasoma urophthalmus* [50]. The lipase activity peaked at 25, 50, and 64 dph. At 25 dph, the digestive tract was well developed, and the success rate of feeding for fry was high, resulting in an increase in food intake. At 50 and 64 dph, the main feed was Artemia and compound feed. These three stages occurred during the period when the fish fry consumed a large amount of feed, so lipase activity showed an upward trend, improving nutrient absorption and promoting fish growth and development.

The activity of acid and alkaline proteases could be detected in the 0 dph larvae, and the activity of former was much lower than that of the latter between 0 and 20 dph. Similar findings were reported in the seabass *Lates calcarifer* [51], tawny puffer *T. flavidus* [52], and tongue Sole *Cynoglossus semilaevis* [53]. These results indicated that the stomach of *T. modestus* is not yet fully developed during these stages, with weak gastric acid secretion and low acid protease activity. Therefore, protein digestion mainly relies on alkaline protease, and higher activity alkaline protease activity could partially compensate for the lack of acidic protease to a certain extent. In this study, the alkaline protease activity significantly increased from 0 to 12 dph, and then decreased between 12 and 25 dph. Similar results were also reported in the *P. Fluviatilis* [54], Persian sturgeon *Acipenser persicus* [55], and *D. Labrax* [9]. Cuvier-Péres et al. [54] reported that the pattern of alkaline protease activity first increasing and then decreasing to a steady state is a clear characteristic of individual development in vertebrates, including fish, and is an important step in the process of metamorphosis.

The activity of acid phosphatase showed a decreasing trend during the early development stage in *T. modestus*, and similar changes were observed in Senegal sole *Solea senegalensis* [56]. Moreover, in this study, we found that the alkaline phosphatase activity was higher than that of acid phosphatase at all stages of early development in *T. modestus*, suggesting that alkaline phosphatase plays a more important role in early development.

### 4.4. Effects of Growth-Related Hormones on Growth and Development

At 0 dph, T3 and T4 can already be detected in *T. modestus*, but the levels are relatively low. Similar results were found in the black rockfish *Sebastes schlegelii* [57] and *P. olivaceus* [58]. Furthermore, de Jesus et al. [59] reported that for most marine fish, the thyroid hormones in embryos and newly hatched larvae originate from maternal thyroid hormones and are relatively abundant. In this study, the low concentration of thyroid hormones in newly hatched larvae may be related to the lower degree of thyroid development and the fact that most of the thyroid hormones from the mother are consumed during embryonic development or that the thyroid hormones from the mother are maintained for a shorter period of time. Throughout the early process in *T. modestus*, the T4 content was much higher than that of T3, and the change trends of the two were very similar. This is consistent with research findings in the rabbitfish *Siganus guttatus* [60], brown spotted grouper *E. tauvina* [61], and *T. modestus* [62]. The reason may be the existence of 5′-monodeiodinase in the body, which can convert a portion of T4 into T3 through deiodination in peripheral tissues.

The GH content of *T. modestus* shows a general upward trend, indicating a positive correlation between GH content and fish development. GH briefly decreases from 57 to 64 dph, possibly due to the developmental stage during which various organs undergo drastic changes and fish fry grow slowly. After 64 dph, GH continues to rise, further indicating a positive correlation between GH and fish growth and development [63]. The IGF-I contents significantly increased from 0 to 12 dph and then significantly decreased from 12 to 35 dph, possibly due to the drastic changes in the development of various organs and digestive systems in juvenile fish during this period. From 35 to 91 dph, IGF-I showed a trend of increase–decrease–increase. During this period, various growth indicators of fish increased, and the increase in IGF-I and GH content enhanced the feeding activity of fish fry and promoted their growth. After 57 dph, the IGF-I content began to decrease, which may be because the juveniles are in a critical period of growth and development at this stage. To adapt to this physiological change, the endocrine system of fish adjusts the secretion of hormones, which are crucial in organ development, while reducing the synthesis and secretion of IGF-I. This finding is consistent with the report by Zhai et al. [64] that thyroid hormone in flounder can exert a certain inhibitory effect on growth by regulating the expression of IGF-binding protein mRNA and downregulating the expression of IGF mRNA. This series of hormone regulation collectively affects the growth and development process in *T. modestus*.

## 5. Conclusions

In summary, we speculated that the gonads had differentiated into ovaries or testes at 60 dph in *T. modestus*. The significantly increased E2 and T levels at 60 dph may, respectively, induce the gonadal differentiation in female and male *T. modestus*. The activity of six digestive enzymes could be detected in newly hatched larvae, indicating that the larvae already have the ability to digest food when transitioning from endogenous to exogenous nutrition. Furthermore, T4 may play more important roles than T3. The levels of IGF-I and GH are positively correlated with development in fish, indicating that IGF-I and GH jointly promote fish growth.

Since the global output and quality of *T. modestus* are gradually decreasing, these results provide useful information for promoting the growth, artificial reproduction, and sex regulation in this species. Further studies are essential for understanding the regulatory mechanisms from hatching to maturation in *T. modestus*, aimed at cultivating this species better.

## Figures and Tables

**Table 1:** Body weight, total length, and body length at each age in *Thamnaconus modestus*.

Days Post-Hatching	Sampling Number	Body Weight (mg)	Total Length (mm)	Body Length (mm)
10	30	0.45 ± 0.09 ^a^	3.11 ± 0.17 ^a^	2.90 ± 0.16 ^a^
15	30	0.62 ± 0.11 ^a^	4.62 ± 0.15 ^a^	3.71 ± 0.16 ^a^
20	30	2.90 ± 0.62 ^a^	5.71 ± 0.23 ^a^	4.42 ± 0.21 ^a^
25	30	41.10 ± 9.89 ^b^	11.39 ± 0.73 ^b^	10.07 ± 0.56 ^b^
30	30	113.33 ± 15.80 ^c^	19.20 ± 1.16 ^c^	17.27 ± 0.90 ^c^
35	30	225.67 ± 97.10 ^d^	33.21 ± 1.14 ^d^	30.21 ± 0.84 ^d^
40	30	644.21 ± 151.11 ^e^	41.81 ± 0.97 ^e^	40.20 ± 0.90 ^e^
45	30	1014.42 ± 286.08 ^f^	45.40 ± 3.11 ^f^	44.28 ± 2.70 ^f^
50	30	1356.86 ± 461.81 ^g^	51.97 ± 3.61 ^g^	49.89 ± 2.83 ^g^
55	30	1869.34 ± 461.81 ^h^	57.53 ± 5.46 ^h^	55.23 ± 5.33 ^h^
60	30	2460.56 ± 109.89 ^i^	62.19 ± 0.05 ^i^	60.04 ± 0.05 ^i^

Note: The data is shown as means ± SD. The different lowercase letters within the same column indicate significant differences (*p* < 0.05), while labels with the same or no letters indicate no significant differences (*p* > 0.05).

## Data Availability

The data that support the findings of this study are available from the corresponding author upon reasonable request.

## Supplementary Materials

The following supporting information can be downloaded at: https://www.mdpi.com/article/10.3390/biology14101385/s1, File S1: Materials and Methods. Reference [28] is cited in the Supplementary Materials.
