# Supplementary material for: Changes in Gonadal Sex Differentiation, Digestive Enzymes, and Growth-Related Hormone Contents in the Larval and Juvenile Black Scraper, Thamnaconus modestus
Source: Biology (Basel). 2025 Oct 10;14(10):1385. doi: 10.3390/biology14101385 (PMC12561227; doi:10.3390/biology14101385)
Supplement: Supplementary file 1 [file biology-14-01385-s001.zip › biology-3900247-supplementary.pdf]

Supplementary

2.2.1 Observation of gonadal histology

The samples stored in Bouin's solution were subjected to stepwise dehydration treatment with alcohol concentration gradients of 80%, 85%, 90%, 95%, and 100% and then fixed on wax blocks for sectioning (thickness 5–8 μm) after transparent xylene and paraffin embedding. This was followed by dewaxing with xylene, rehydration with gradient alcohol, staining with hematoxylin and eosin, rendering them transparent with xylene, and sealing with neutral gum. We observed and photographed the samples using an Olympus FX380 microscope (Olympus Corporation, Tokyo, Japan).

2.2.2 Determination of sex hormone content

The levels of E2 and T were determined using an enzyme-linked immunosorbent assay as described by our previous study [29]. The serum sample frozen at –70 °C was thawed at 4 °C and centrifuged again to obtain the upper layer serum. Then, the serum was purified for steroids using a methanol ether low-temperature freeze evaporation system. The serum sample obtained after 3 rounds of freeze evaporation could be used for detection. The E2 and T concentrations were quantified using an Estradiol EIA kit or a Testosterone EIA kit (Cayman Chemical, Ann Arbor, MI, USA) according to the manufacturer's instructions.

2.2.3 Determination of digestive enzyme activity and hormone content

The whole homogenate of fish fry at 0–25 dph, the abdomen of fry at 30 and 35 dph, and a certain proportion of physiological saline were added, homogenized under ice–water bath conditions, and then placed in a freeze centrifuge. After centrifugation at 4 °C and 2500 r/min for 10 minutes, the supernatant was taken, to measure the activity of various digestive enzymes.

The amylase and lipase activities were measured using amylase and lipase assay kits (Shanghai Jiwei Biotechnology Co., Ltd., Shanghai, China), respectively. The activities of acid and alkaline proteases were detected using acidic and alkaline proteinase assay kits (Shanghai Jiwei Biotechnology Co., Ltd., Shanghai, China), respectively. The activities of acid and alkaline phosphatases were also detected using acid and alkaline phosphatase assay kits (Shanghai Jiwei Biotechnology Co., Ltd., Shanghai, China), respectively.

The hormone content was determined using the fish triiodothyronine (T3), thyroxine (T4), GH, and IGF-1 ELISA Kit from Wuhan Huamei Biotechnology Co., Ltd. (Wuhan, China), performing an enzyme-linked immunosorbent assay (ELISA) according to the manufacturer's instructions.
